# Transplant Recipients Using Tacrolimus Had Higher Utilization of Healthcare Services Than Those Receiving Cyclosporine in Taiwan

**DOI:** 10.3389/fphar.2019.01074

**Published:** 2019-09-19

**Authors:** Yi-Chang Lin, Chien-Sung Tsai, I-Hsun Li, Yi-Ting Tsai, Tien-Yu Huang, Kwai-Fong Lee, Chih-Sheng Lin, Jui-Hu Shih, Li-Ting Kao

**Affiliations:** ^1^Department of Biological Science and Technology, National Chiao Tung University, Hsinchu, Taiwan; ^2^Division of Cardiovascular Surgery, Department of Surgery, Tri-Service General Hospital, National Defense Medical Center, Taipei, Taiwan; ^3^Department of Pharmacy Practice, Tri-Service General Hospital, Taipei, Taiwan; ^4^School of Pharmacy, National Defense Medical Center, Taipei, Taiwan; ^5^Division of Gastroenterology, Department of Internal Medicine, Tri-Service General Hospital, National Defense Medical Center, Taipei, Taiwan; ^6^Biobank Management Center, Tri-Service General Hospital, Taipei, Taiwan; ^7^School of Public Health, National Defense Medical Center, Taipei, Taiwan; ^8^Graduate Institute of Life Sciences, National Defense Medical Center, Taipei, Taiwan

**Keywords:** healthcare service utilization, tacrolimus, cyclosporine, heart transplant, kidney transplant, liver transplant

## Abstract

To date, population-based studies on the healthcare service utilization among stable heart, kidney, and liver transplant recipients with different calcineurin inhibitors are still scarce. Therefore, we used the Taiwan National Health Insurance Research Database to conduct a nationwide cross-sectional study to estimate the healthcare utilization of stable transplant recipients with tacrolimus or cyclosporine (n = 3,482). The sampled patients in this study comprised 377 heart, 1,693 kidney, and 1,412 liver transplant recipients between 1 January 2011 and 31 December 2011. Each subject was followed for a 1-year period to evaluate his/her healthcare service utilization. Outcome variables of the healthcare service utilization were stated as below: numbers of outpatient visits, outpatient costs, numbers of inpatient days, inpatients costs, and total costs of all healthcare services. As for all healthcare service utilization, stable transplant recipients on tacrolimus had significantly more outpatient visits (40.7 vs. 38.6), outpatient costs (US$10,383 vs. US$8,155), and total costs (US$12,516 vs. US$10,372) of all healthcare services than those on cyclosporine during the 1-year follow-up period. Additionally, further analysis showed that heart transplant recipients receiving tacrolimus incurred 1.7-fold higher inpatient costs compared to patients receiving cyclosporine. We concluded that transplant recipients using tacrolimus had significantly higher utilization of all healthcare services than those receiving cyclosporine as immunosuppressive therapy.

## Introduction

Solid organ transplantation is expensive and exerts a huge financial burden on transplant recipients. The average reported cost of a solid organ transplant ranges from $334,300 for a single kidney transplant to more than $2.3 million for combined heart and lung transplants (Bentley and Hanson, 2014). The use of immunosuppressive medications after transplantation is essential for the success of organ transplantation because immunosuppressive regimens can reduce acute rejection rates and graft loss resulting from acute rejection (Meier-Kriesche et al., 2004). In the early 1980s, cyclosporine, a calcineurin inhibitor, was introduced in the market and provided excellent renal graft survival rates (Starzl et al., 1980; Gjertson et al., 1995). This agent impairs the transcription of interleukin-2 and several other cytokines in T lymphocytes (Jasiak and Park, 2016). However, cyclosporine may lead to adverse reactions such as nephrotoxicity, chronic hemolytic uremic syndrome, hyperlipidemia, hypertension, gingival hyperplasia, diabetes mellitus, and tremors. Since its Food and Drug Administration (FDA) approval in the early 1990s, tacrolimus has been widely used as an alternative immunosuppressive medication after all types of solid organ transplants; it is a more potent calcineurin inhibitor. Compared with cyclosporine, tacrolimus provides lower acute rejection rates and potentially higher graft survival rates, as demonstrated by several randomized controlled trials and meta-analyses; however, tacrolimus is associated with a higher risk of posttransplant diabetes mellitus (Gjertson et al., 1995; Knoll and Bell, 1999; Webster et al., 2005a). Tacrolimus may plausibly result in additional costs of managing adverse events and increase the non-transplant-related health service utilization of transplant recipients.

Until now, few studies have estimated the healthcare service utilization of stable heart, kidney, and liver transplant recipients receiving different calcineurin inhibitors. Studies have shown that compared with cyclosporine, tacrolimus-based immunosuppression is associated with economic advantages among kidney or liver transplant recipients due to its lower acute rejection rates (Lake et al., 1995; Neylan et al., 1998; Rabkin et al., 2001; Lazzaro et al., 2002; Miners et al., 2007). However, cost-effectiveness analyses in Brazil and Germany have revealed that tacrolimus treatment entails higher total health service expenditures than cyclosporine treatment after renal transplantation (Jurgensen et al., 2010; Guerra Junior et al., 2015). Previous reports have indicated inconsistent economic impacts of calcineurin inhibitors on the healthcare system. In addition, to date, no study has explored healthcare service utilization in heart transplant recipients receiving calcineurin inhibitors. Thus, this population-based study investigated the differences in healthcare service utilization among stable heart, kidney, and liver transplant recipients receiving cyclosporine or tacrolimus-based treatment during a 1-year follow-up period in Taiwan.

## Materials and Methods

### Database

This study used data of the de-identified medical claims from the Taiwan National Health Insurance Research Database (NHIRD). This database includes medical records and registry files for approximately 99% of the Taiwanese population (*n* = 23 million) since 1995. The NHIRD has been released to investigators in Taiwan for research purposes, and researchers are permitted to track the longitudinal records of the enrollees. The need for ethics approval was waived by the Tri-Service General Hospital Institutional Review Board because it only used de-identified secondary data. The data sets for this manuscript are not publicly available, because the data are handled and stored by the Health and Welfare Data Science Center (HWDC). Requests to access the data sets should be directed to the HWDC, Department of Statistics, Ministry of Health and Welfare, Taiwan (http://dep.mohw.gov.tw/DOS/np-2497-113.html).

### Study Sample

In this nationwide cross-sectional study, 3,902 transplant recipients (including heart, kidney, and liver transplant recipients) receiving cyclosporine or tacrolimus between 1 January 2011 and 31 December 2011 were first identified. The use of the study drug was determined on the basis of the Anatomical Therapeutic Chemical codes (L04AD01 for cyclosporine and L04AD02 for tacrolimus). Generally, given that acute rejection mostly occurs within weeks to 1 year after transplantation, the index date was defined as the last date on which patients received cyclosporine or tacrolimus during the study period to minimize the acute rejection factor. To ensure equal follow-up periods among all selected stable transplant recipients, we excluded 175 patients who died within the study period after the index date. We further excluded 245 patients aged <18 years to ensure that adult transplant recipients were recruited. Accordingly, 3,482 transplant recipients receiving cyclosporine or tacrolimus were recruited into this study. In addition, 2,741 tacrolimus users were identified as the study group, and 741 cyclosporine users were defined as the comparison group. We further divided the study patients into three groups: heart transplant recipients, kidney transplant recipients, and liver transplant recipients.

### Variables of Interest

In order to carry out the healthcare service utilization assessments and evaluate patients’ healthcare service visits and costs, all transplant recipients in this study were tracked for 1 year following the index date. Healthcare service in this study included those of physician diagnoses, medications, surgery, and laboratory tests covered by National Health Insurance (NHI) program. Variables of outpatient service utilization during the 1-year follow-up period in this study were defined as follows: 1) mean numbers of outpatient visits, 2) mean total costs of outpatient services, 3) mean costs of outpatient study drugs (cyclosporine and tacrolimus), and 4) mean costs of other outpatient services (excluding costs of study drugs to avoid the effects due to different drug prices between cyclosporine and tacrolimus). Variables of inpatient service utilization were identified as follows: 1) mean numbers of inpatient days, 2) mean total costs of inpatient services, 3) mean costs of inpatient study drugs, and 4) mean costs of other inpatient services (excluding costs of study drugs to avoid the effects due to different drug prices between cyclosporine and tacrolimus). Additionally, variables of all NHI healthcare services’ costs were defined as follows: 1) mean total costs, 2) mean costs of study drugs, and 3) mean costs of other healthcare services (excluding costs of study drugs to avoid the effects due to different drug prices between cyclosporine and tacrolimus).

### Statistical Analysis

Pearson chi-squared tests were conducted to compare the differences in sex, urbanization level (five levels, with 1 being the most urbanized and 5 being the least urbanized), monthly income, transplanted organs, and comorbidities between cyclosporine and tacrolimus users (Liu et al., 2006). Independent *t* tests were performed to compare the differences in the utilization and costs between cyclosporine and tacrolimus users. A two-sided *p* value of <0.05 was used to evaluate statistical significance. All statistical analyses were performed using the SAS system (version 9.4, SAS System for Windows).

## Results

This study included a total of 3,482 stable transplant recipients who received tacrolimus or cyclosporine. Among the sampled transplant recipients in this study, 2,741 were defined as tacrolimus users, and 741 were identified as cyclosporine users. The demographic characteristics and comorbidities of the sampled patients are presented in **Table 1**. The mean age of tacrolimus and cyclosporine users was 50.2 ± 11.6 and 50.2 ± 11.4 years, respectively (p = 0.912). Although the mean number of years after transplantation was statistically fewer in tacrolimus users (3.7 ± 2.9 years) than in cyclosporine users (4.9 ± 3.2 years), the time after transplantation was sufficiently long to consider these recipients as being relatively stable. Other findings revealed that the distributions of sex, hypertension, hyperlipidemia, coronary heart disease, arrhythmia, thyroid disease, anxiety, and transplanted organ were significantly different between tacrolimus and cyclosporine users. However, no significant difference was observed in age, urbanization level, monthly income, and diabetes between the two groups.

**Table 1:** Demographic characteristics of tacrolimus and cyclosporine users (*N* = 3,482).

Variable	Tacrolimus users (*n* = 2,741)		Cyclosporine users (*n* = 741)		*p* value
	Total no.	%	Total no.	%	
Age (years)	50.2 ± 11.6		50.2 ± 11.4		0.912
Sex					0.001
Male	1,716	62.6	413	55.7	
Female	1,025	37.4	328	44.3	
Urbanization level					0.050
1 (most urbanized)	836	30.5	262	35.4	
2	810	29.6	199	26.9	
3	454	16.6	125	16.9	
4	361	13.2	77	10.4	
5 (least urbanized)	280	10.2	78	10.5	
Monthly income					0.548
US$0–US$600	1,104	40.3	304	41.0	
US$601–US$1,200	1,054	38.5	293	39.5	
≥US$1,201	583	21.3	144	19.4	
Transplanted organ					<0.001
Heart	208	7.6	169	22.8	
Kidney	1,254	45.8	439	59.2	
Liver	1,279	46.7	133	18.0	
Year after transplantation	3.7 ± 2.9		4.9 ± 3.2		<0.001
Comorbidities					
Hypertension	1,043	38.1	206	27.8	<0.001
Hyperlipidemia	1,699	62.0	428	57.8	0.036
Diabetes	1,843	67.2	481	64.9	0.233
Coronary heart disease	1,985	72.4	465	62.8	<0.001
Arrhythmia	2,214	80.77	502	67.75	<0.001
Thyroid disease	1,020	37.21	403	54.39	<0.001
Anxiety	1,345	49.07	264	35.63	<0.001

**Table 2** presents the utilization and costs of outpatient services, inpatient services, and all healthcare services within the 1-year study period following the index date for transplant recipients receiving cyclosporine or tacrolimus. Regarding outpatient service utilization, tacrolimus users had a significantly higher number of outpatient visits (40.7 vs. 38.6) and total outpatient costs (US$10,383 vs. US$8,155) than cyclosporine users. However, no significant difference was observed in inpatient days and total inpatient costs between tacrolimus and cyclosporine users. Additionally, regarding all healthcare services, tacrolimus users had higher total costs (US$12,516 vs. US$10,372) than cyclosporine users.

**Table 2:** Utilization and costs of outpatient services, inpatient services, and all healthcare services within 1 year for tacrolimus and cyclosporine users (*N* = 3,482).

Variable	Tacrolimus users (*n* = 2,741)		Cyclosporine users (*n* = 741)		*p* value
	Mean	SD	Mean	SD	
Outpatient services					
Outpatient visits (no.)	40.7	19.3	38.6	18.9	0.009
Total outpatient costs (US$)	10,383	9,825	8,155	4,609	<0.001
Inpatient services					
Inpatient days (no.)	7.4	21.7	8.2	19.4	0.332
Total inpatient costs (US$)	2,133	6,952	2,217	6,192	0.749
All NHI healthcare services					
Total costs (US$)	12,516	12,459	10,372	8,562	<0.001

SD, standard deviation.

NHI, National Health Insurance

**Table 3** presents the total health service utilization, outpatient service utilization, and inpatient service utilization within 1 year for tacrolimus and cyclosporine users among heart, kidney, and liver transplant recipients. Among heart transplant recipients, tacrolimus users had a significantly higher number of outpatient visits (37.3 vs. 32.2), total outpatient costs (US$11,991 vs. US$9,407), outpatient study drug costs (US$4,197 vs. US$2,394), total inpatient costs (US$4,729 vs. US$2,737), inpatient study drug costs (US$202 vs. US$119), and other inpatient costs (US$4,527 vs. US$2,618) than cyclosporine users. Regarding all healthcare service utilization among heart transplant recipients, tacrolimus users had significantly higher total costs (US$16,720 vs. US$12,144), study drug costs (US$4,399 vs. US$2,513), and other costs (US$12,321 vs. US$963) than cyclosporine users.

**Table 3:** Utilization and costs of healthcare services within 1 year for cyclosporine and tacrolimus users, stratified by different transplantations.

Variable	Heart transplant recipients (*n* = 377)			Kidney transplant recipients (*n* = 1,693)			Liver transplant recipients (*n* = 1,412)		
	Tacrolimus users (*n* = 208)	Cyclosporine users (*n* = 169)	*p* value	Tacrolimus users (*n* = 1,254)	Cyclosporine users (*n* = 439)	*p* value	Tacrolimus users (*n* = 1,279)	Cyclosporine users (*n* = 133)	*p* value
	Mean (SD)			Mean (SD)			Mean (SD)		
Outpatient services									
Outpatient visits (no.)	37.3 (19.8)	32.2 (16.0)	0.005	41.0 (18.9)	40.5 (18.6)	0.624	40.8 (19.5)	40.3 (21.5)	0.767
Total outpatient costs (US$)	11,991 (5,997)	9,407 (5,131)	<0.001	10,378 (12,726)	7,978 (4,182)	<0.001	10,125 (6,471)	7,146 (4,939)	<0.001
Study drug costs	4,197 (3,091)	2,394 (1,056)	<0.001	4,003 (2,797)	2,120 (1,574)	<0.001	3,321 (2,463)	1,995 (1,054)	<0.001
Other outpatient costs	7,794 (4,989)	7,013 (5,107)	0.136	6,375 (12,563)	5,858 (4,091)	0.202	6,805 (5,694)	5,151 (4,777)	<0.001
Inpatient services									
Inpatient days (no.)	11.7 (27.4)	10.3 (23.6)	0.591	6.2 (18.0)	6.9 (16.3)	0.415	7.8 (23.9)	9.6 (22.6)	0.422
Total inpatient costs (US$)	4,729 (10,403)	2,737 (5,286)	0.017	1,735 (6,248)	2,011 (6,779)	0.453	2,102 (6,823)	2,237 (5,136)	0.780
Study drug costs	202 (379)	119 (298)	0.018	66 (202)	45 (145)	0.016	94 (360)	84 (254)	0.686
Other inpatient costs	4,527 (10,219)	2,618 (5,035)	0.019	1,668 (6,113)	1,967 (6,696)	0.412	2,008 (6,613)	2,153 (4,970)	0.757
All NHI healthcare services									
Total costs (US$)	16,720 (12,203)	12,144 (7,924)	<0.001	12,113 (14,770)	9,989 (9,010)	<0.001	12,227 (9,574)	9,384 (7,499)	<0.001
Study drug costs	4,399 (3,238)	2,513 (1,180)	<0.001	4,070 (2,829)	2,164 (1,604)	<0.001	3,415 (2,523)	2,079 (1,140)	<0.001
Other costs	12,321 (11,763)	9,631 (7,650)	0.008	8,044 (14,703)	7,825 (9,002)	0.714	8,812 (8,996)	7,304 (7,240)	0.027

SD, Standard deviation.

Among kidney transplant recipients (**Table 3**), tacrolimus users had higher total costs and study drug costs for outpatient services and all healthcare services than cyclosporine users. In addition, tacrolimus users had higher inpatient study drug costs than cyclosporine users. **Table 3** also shows the utilization of liver transplant recipients. The findings revealed that tacrolimus users had higher total costs (US$10,125 vs. US$7,146), study drug costs (US$3,321 vs. US$1,995), and other costs (US$6,805 vs. US$5,151) for outpatient services than cyclosporine users. Furthermore, regarding all healthcare services, tacrolimus users had higher total costs (US$12,227 vs. US$9,384), study drug costs (US$3,415 vs. US$2,079), and other costs (US$8,812 vs. US$7,304) than cyclosporine users.

## Discussion

This population-based study found that stable transplant recipients receiving tacrolimus had higher healthcare service utilization, including outpatient visits, outpatient costs, and total costs of all NHI healthcare services, than transplant recipients receiving cyclosporine (**Table 2**). The higher outpatient visits among tacrolimus users than among cyclosporine users may be caused by the high prevalence of some chronic diseases, including hypertension, hyperlipidemia, and coronary heart disease, in tacrolimus users (**Table 1**). A possible explanation is that tacrolimus might be preferred for patients with hypertension, sodium retention, and hypercholesterolemia. Additionally, cyclosporine might be the more favorable choice for patients with diabetes mellitus, and it has milder neurological side effects (Danovitch, 1997; Pirsch et al., 1997; Webster et al., 2005a; Haddad et al., 2006). Therefore, stable transplant recipients receiving tacrolimus may experience more comorbidities, including hypertension, hyperlipidemia, and coronary heart disease, than cyclosporine users, thereby resulting in more outpatient visits. Moreover, no difference was observed in the distribution of diabetes mellitus between tacrolimus and cyclosporine users (**Table 1**), although previous studies have found that patients receiving tacrolimus had a higher incidence of *de novo* insulin-requiring diabetes mellitus (Webster et al., 2005a; Haddad et al., 2006). This finding might be explained reasonably by the fact that cyclosporine is more commonly prescribed for transplant recipients with diabetes mellitus. Furthermore, unsurprisingly, outpatient study drug costs in tacrolimus users were higher than those in cyclosporine users in this study due to the higher actual wholesale price of tacrolimus (James and Mannon, 2015). Our findings are in agreement with those of previous economic studies indicating that regimens containing cyclosporine were more cost-effective than tacrolimus-based regimens in renal transplant recipients (Guerra Junior et al., 2010; Jurgensen et al., 2010; Guerra Junior et al., 2015).

In the United States, previous studies comparing cyclosporine with tacrolimus in renal transplant recipients concluded that tacrolimus use led to higher cost savings because of the lower rates of hospitalization as a result of fewer acute rejection episodes (James and Mannon, 2015; Kamel et al., 2016). However, our study found no difference in days and total costs of inpatient services between stable transplant recipients receiving tacrolimus and those receiving cyclosporine in Taiwan (**Table 2**). It is reasonably speculated that a similar rate of graft survival was observed among stable transplant recipients regardless of the use of different study drugs after the acute rejection period (Opelz, 1999; Orme et al., 2003; Webster et al., 2005b). In Brazil, Gomes et al. showed poorer graft survival rates (survival rates of 64.8% and 71.9%, respectively) and increased risk of death or return to dialysis (hazard ratio = 1.194; 95% CI 1.082–1.318) in patients treated with tacrolimus compared with those treated with cyclosporine after a 10-year follow-up (Gomes et al., 2016). Therefore, different ethnicities may contribute to consistent outcomes from these studies.

To the best of our knowledge, this is the first study to provide a real-world assessment of healthcare service utilization, including complete utilization information and medical costs, among stable heart transplant recipients receiving calcineurin inhibitors through analysis of the nationwide population-based data set NHIRD. To date, studies on healthcare service utilization among heart transplant recipients receiving different calcineurin inhibitors are limited. Studies have indicated that heart transplant recipients treated with cyclosporine presented higher rates of cytomegalovirus infection (Rodriguez-Serrano et al., 2014), thereby increasing the need for treatment by approximately eightfold (Bond et al., 2018), than those treated with tacrolimus. Compared with tacrolimus as baseline immunosuppressive therapy, cyclosporine may also produce higher risks of obesity (Lopez-Vilella et al., 2015) and a more pronounced deterioration of renal function (Helmschrott et al., 2015) after heart transplantation. The abovementioned side effects of cyclosporine may be a key factor for the increase in healthcare service utilization. However, among stable heart transplant recipients, we found that tacrolimus users had approximately five more outpatient visits than cyclosporine users. Regarding inpatient services, although no difference was observed in the number of inpatient days between heart transplant recipients receiving tacrolimus and those receiving cyclosporine, tacrolimus users had approximately 1.7-fold higher total inpatient costs, especially nonstudy drug–related costs, than cyclosporine users (**Table 3**).

Several limitations of this study should be considered. First, this study did not evaluate self-paid healthcare services (such as over-the-counter medicines) and indirect costs in transplant recipients. Secondly, many undocumented factors may potentially affect the utilization and costs of healthcare services in this study. Third, the study findings should be generalized to other ethnicities with discretion because most of the patients included in this study were Chinese Han.

In conclusion, this population-based study revealed that stable transplant recipients receiving tacrolimus had higher outpatient healthcare service utilization than those receiving cyclosporine. Additionally, heart transplant recipients receiving tacrolimus had approximately 1.7-fold higher total costs of inpatient healthcare services than those receiving cyclosporine, but no difference was noted in inpatient days within the 1-year study period following the index date between transplant recipients receiving cyclosporine and those receiving tacrolimus. Based on the study results, we suggest that physicians should consider the economic impact of tacrolimus on lower-income stable transplant recipients, especially heart transplant recipients. Additional studies should be conducted to further investigate the potential factors leading to elevated healthcare service utilization by stable transplant recipients receiving tacrolimus.

## Data Availability

The datasets for this manuscript are not publicly available because the data are handled and stored by the Health and Welfare Data Science Center (HWDC). Requests to access the datasets should be directed to the HWDC, Department of Statistics, Ministry of Health and Welfare, Taiwan (http://dep.mohw.gov.tw/DOS/np-2497-113.html).

## Ethics Statement

This study was approved by the Institutional Review Board of Tri-Service General Hospital.

## Author Contributions

C-SL, J-HS, and L-TK conceived of this study and participated in study design. Y-CL wrote and revised the manuscript. C-ST and I-HL provided guidance and edited the paper. Y-TT and T-YH participated in the performance of the research. K-FL participated in data analysis. L-TK performed statistical analysis and supervised the entire project. All authors critically reviewed content and approved the final version for publication.

## Conflict of Interest Statement

The authors declare that the research was conducted in the absence of any commercial or financial relationships that could be construed as a potential conflict of interest.
